# Research on drinking-groundwater source safety management based on numerical simulation

**DOI:** 10.1038/s41598-020-72520-7

**Published:** 2020-09-23

**Authors:** Kai Song, Xu Ren, Adam Khalifa Mohamed, Jian Liu, Fei Wang

**Affiliations:** Faculty of Geosciences and Environmental Engineering, Southwest Jiaotong University, No. 111, North Section 1, 2nd Ring Road, Chengdu, 610031 China

**Keywords:** Environmental impact, Environmental chemistry, Environmental impact

## Abstract

A drinking-groundwater source protection technology system based on a three-dimensional finite-difference groundwater model was constructed and applied to the safe management of drinking groundwater in the first terrace of Fujiang River. In the study area, the main type of groundwater is the quaternary systemic alluvial deposit loose rock pore water and the aquifer thickness varies between 20 and 35 m. Groundwater is the main source of water and is used for various purposes through two exploitation wells. The water volumes of 1# exploitation well (1#) and 2# exploitation well (2#) are 10,000 m^3^/day and 5000 m^3^/day, respectively. An analysis of 22 indicators from 11 groundwater samples showed that a higher concentration of chemical-oxygen-demand (COD_Mn_) and ammonia–nitrogen (NH_3_–N), and they had a high correlation with most of the other water-quality factors. Therefore, COD_Mn_ and NH_3_–N were selected as indicator factors for model calibration and prediction. Twenty-two hydraulic head observation wells were used for flow-model calibration. The flow model indicated that a drop funnel formed with a maximum depth of 12 m, and the particle-capture zone in the original downstream direction of the south side extended to 1100 m because of groundwater exploitation. The solute-transport model showed that industrial pollution sources were the main factors that led to a deterioration of water quality. To analyze the necessity and effectiveness of remediation measures for the safety of drinking-water sources, two scenarios were considered to predict the concentration of NH_3_-N and COD_Mn_ in groundwater exploitation wells over 20 years. Scenario I, which considered that current conditions were maintained, predicted that the NH_3_-N would exceed the drinking-water quality standard of 0.5 mg/L after 16 years. Scenario II, in which industrial sewage treatment plants were installed outside the particle-trapped zone of the exploitation wells and strict anti-seepage measures were implemented, predicted that the peak concentrations of NH_3_-N and COD_Mn_ in the exploitation wells would be 0.26 mg/L and 1.33 mg/L, respectively, after 3 years of model operation. This study provides a theoretical basis for drinking-groundwater source protection that can be applied to safety management practices.

## Introduction

Groundwater is an integral part of the hydrological cycle and an important source of drinking water, but its pollution was triggered by different anthropogenic activities has become a global concern^1–3^. The rapid expansion of anthropogenic activities has become a major cause of pollutant dispersion in the subsurface environment, and the deterioration of groundwater quality is not uncommon^4–6^. Despite differences in the degree of economic development and environmental protection in various regions, the protection of drinking-water sources should be the primary focus for human health.

The Fujiang River is an important tributary in the upper reaches of the Yangtze River Basin. This area belongs to the Yangtze River Green Circular Economic Belt planned by the Chinese government and is the main remediation area for environmental issues. The issues in the study area are a microcosm of the current state of the environment that has been caused by industrial development and rapid urbanization in China. In the same hydrogeological unit, there is a contradiction that such as an unreasonable urban spatial layout, anthropogenic engineering intervention has led to a deterioration in groundwater quality and the groundwater demand for industrial, agricultural and domestic use has resulted in a continued increased consumption. Several water-quality and pollution-source-distribution studies have been conducted on the Fujiang River terrace. The survey found that more than 10% of major industrial-pollution sources yield excessive emissions, and organic pollution is the main pollution in the Fujiang River terrace^7^. Among various contaminants, the chemical-oxygen-demand (COD_Mn_) and ammonia–nitrogen (NH_3_-N) emissions need to be controlled strictly to protect water resources^8^. According to statistics, the Fujiang River terrace distributes dozens of drinking-groundwater sources. However, no previous studies have been conducted on the drinking-groundwater source safety risk caused by rapid urbanization and more frequent anthropogenic engineering activities in the study area.

Groundwater modeling is an excellent method to analyze groundwater resource management issues such as sustainable exploitation and pollution, which can representational present conceptual model of groundwater system with the various hydrogeological situation. The groundwater model visualizes complex groundwater systems. When the rationality of the past behavior reproduced by the model is verified by actual monitoring information, they can predict future trends of groundwater to support decision-making and optimize management methods^9,10^. Accurately simulate and predict fluctuations in groundwater levels and quality is the focus of effective management of groundwater^11,12^. Studies on groundwater modeling have been carried out to evaluate the sustainable exploitation of groundwater resource, contaminant migration trends and vulnerability assessment^13–18^. Few studies have been conducted using groundwater numerical models to construct a drinking-groundwater source safety-management system with an input-feedback-decision section.

Taking this into consideration, we developed a fresh drinking-groundwater source safety-management system based on modeling. In this system, the groundwater environment status and response measures are simulated through modeling to provide theoretical support for management decision-making. The system was applied to the protection of drinking-groundwater sources in the Fujiang River terrace with COD_Mn_ and NH_3_-N as the indicator factors to provide practical significance for decision-making in groundwater management.

## Study area

**Figure 1:**
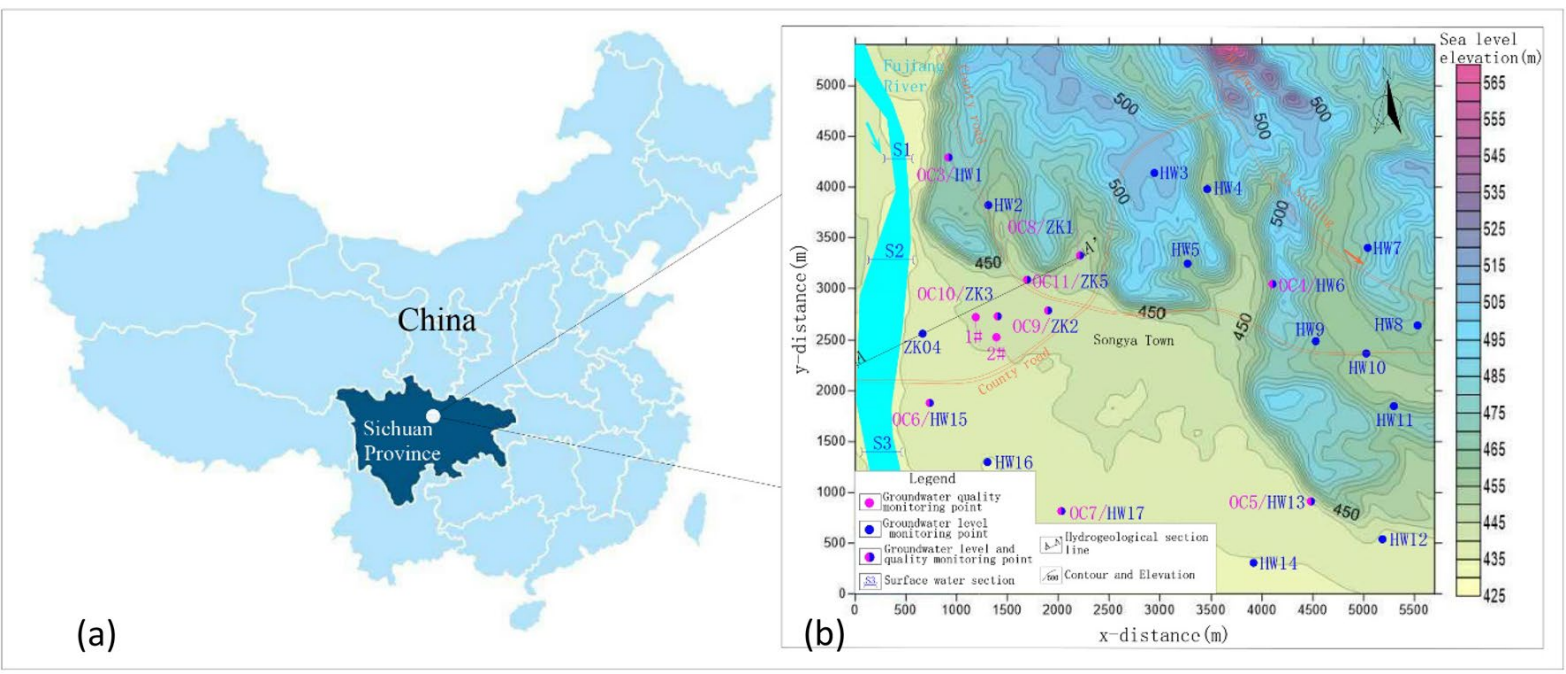
(**a**) Location of the study area, and (**b**) topography and distribution map of monitoring points. (**a**) was generated by AutoCAD version 2014; (**b**) was generated by Surfer version 9.6, according to the data obtained from the hydrogeological survey for this study, the URL of the source map is https://geocloud.cgs.gov.cn/#/portal/geologicalDatabase/ArealGeology?type=dzsjk.

The study area is located in the northwest of the Sichuan basin, which is in the upper reaches of the Fujiang River terrace as shown in Fig. 1. The area is approximately 146 km from Chengdu City, which is the capital of Sichuan, China (Fig. 1), and is located between 30° 42′ to 33° 03′ N and 103° 45′ to 105° 43′ E, and covers an area of 25 km^2^. The study area lies in a subtropical humid monsoon climate. The average annual precipitation is 919 mm, with a maximum annual precipitation of 1700 mm and a minimum of 700 mm. More than 70% of the total precipitation is concentrated in the rainy period from June to September. The mean annual temperature is 10.4 °C, the average temperature in the coldest months is 3.9 to 6.2 °C and the average temperature in the hottest months is 24.2 to 27.2 °C. The annual average relative humidity is 75%. The annual maximum of absolute moisture content is 15.2, with a minimum of 14.3. The average annual sunshine is 1337.5 h. The region is dominated by a northerly wind, with a maximum wind speed of 15.7 m/s and an average wind speed of 1.2 m/s.

### Hydrogeology

The foundation of the hydrogeological conceptual model is the correct identification and cognition of hydrogeological conditions, which can be achieved through field investigation and hydrogeological exploration. The study area has a flat topography with a gentle slope of less than 5%, which belongs to a typical river alluvial landform. The terrace elevation varies from 580 m in the upstream areas to 425 m outside the particle-capture zone of exploitation wells. From hydrogeological exploration results, the main type of groundwater is the quaternary systemic alluvial pore water. In the vertical direction, the terrace medium has obvious dual structural features, which means that the particle size of the medium in the unsaturated zone increases gradually and the main composition changes from clay and fine sand to pebbles from the surface to the depth. Drilling and screening tests show that the proportion of pebbles in the aquifer is 40–60% and the void between pebbles is filled by medium coarse sand. According to hydrogeological pumping tests, the unit water output of the aquifer is 0.39–1.68m^3^/h·m and the hydraulic conductivity is between 10 and 40 m/day. The underlying bedrock of Quaternary systemic alluvial layer is the Mesozoic Cretaceous Jiange Formation (K_1_jn) of which the main component is mudstone cemented by clay minerals. The hydraulic conductivity of bedrock is between 0.01 and 0.1 m/day as determined by a hydrogeological water-pressure test. The significant differences in magnitude of the hydraulic conductivity separate the two types of media into aquifer and aquitard.

**Figure 2:**
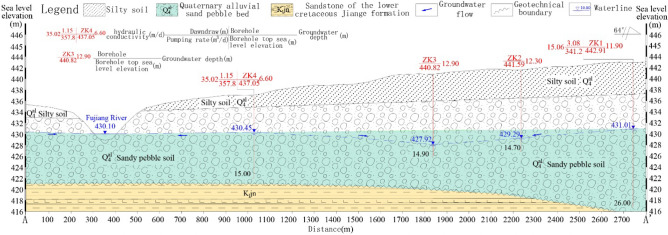
Hydrogeological section of the study area (orientation: landscape 1:5000; portrait 1:200).

Precipitation infiltration is the primary source of potential recharge to the quaternary systemic alluvial aquifer at any period. The main discharge is affected by the degree of groundwater exploitation and changes from discharge streams to artificial exploitation. Without a centralized pumping-well operation period, the groundwater is drained from the northeast to the southwest to the Fujiang River as the lowest erosion reference boundary. After operation, the Fujiang River was transformed from discharge boundary to an intensified recharge source in the study area. As shown by Fig. 2, the basic structure of the aquifer, where the blue dotted line is the hydraulic head level that is determined according to the borehole observation. The values of groundwater head display that the groundwater flow direction changes to the center of the drop funnel and the blue arrow indicates the groundwater flow direction.

### Groundwater exploitation and distribution of pollution sources

**Figure 3:**
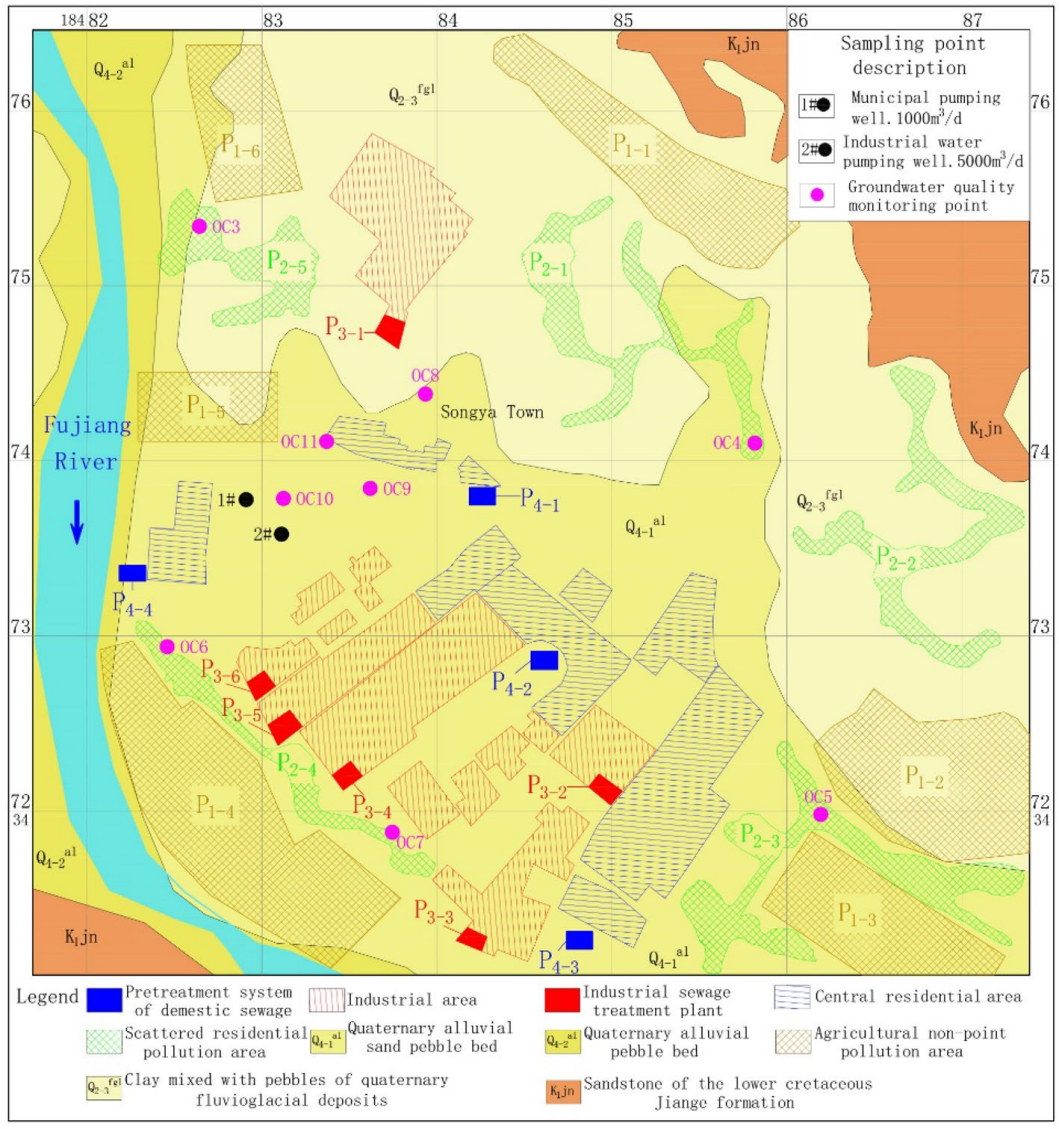
Spatial distribution of exploitation wells and pollution sources. The map was generated by AutoCAD version 2014, according to the data obtained from the investigation of hydrogeology and pollution sources for this study, the URL of the source map is https://geocloud.cgs.gov.cn/#/portal/geologicalDatabase/ArealGeology?type=dzsjk.

Before the 2000s, there was a low density of residents in the area, and most of the land was used for agricultural activity. An increase in population density and frequent industrial activity resulted in two centralized water supply plants being built in the study area until 2010. Rapid growth in water demand reflects the speed of urbanization and population increase. Groundwater is pumped from two exploitation wells as the main source of water for drinking and industrial production. The 1# exploitation well (1#) is located 750 m east of the Fujiang River and the exploitation capacity is 10,000 m^3^/day for human drinking. The 2# exploitation well (2#) is supplied separately to a winery for production, and the exploitation capacity is 5000 m^3^/day. The well is close to 150 m on the southeast side of 1#. The initial environment around the 1# and 2# exploitation wells is simple and contains mainly agricultural land and scattered residential areas with a low population density. However, the decrease in distance between the water source and the high-density area of the population and the industrial factory leads to the gradual accumulation of drinking-groundwater safety risks annually. The susceptibility of groundwater to pollution is a consequence of a finite combination of different factors that range from the variation in hydrogeological settings and human activities, which together often form a dynamic system^19^. An identification of the existing and controlled sources of pollution for risk control is essential to drinking-groundwater safety management. An investigation of pollution sources allows for a classification into non-point pollution source and point pollution sources for statistical analysis based on pollution source-emission characteristics. Non-point pollution source includes agricultural non-point sources (P1) and unorganized emissions from scattered residents (P2). The agricultural non-point source means that the area where the groundwater pollution originates from agricultural activities includes six areas (P1-1 to P1-6) with a total area of 3.93 km^2^ (Fig. 3). The source of unorganized emission sources is attributed to a lack of centralized disposal systems in scattered residential areas that contain 5 areas (P2-1 to P2-5) with a total area of 1.94 km^2^. Point-source pollution is divided into industrial pollution sources (P3) and the pollution of central-residential areas (P4). Contamination identification shows that the main risk of point-source pollution is seepage caused by damage to the sewage storage and disposal facilities. Pollution of the central residential area originates mainly from domestic sewage-pretreatment systems, and industrial pollution sources originate mainly from industrial sewage-treatment plants. Figure 3 shows six industrial sewage-treatment stations (P3-1–P3-6) with a total volume of 16,800 m^3^ and four domestic sewage-treatment pretreatment systems (P4-1–P4-4) with a total volume of 1400 m^3^. As described previously, 21 potential sources of pollution exist around the water-exploitation well.

## Materials and methods

### System construction

**Figure 4:**
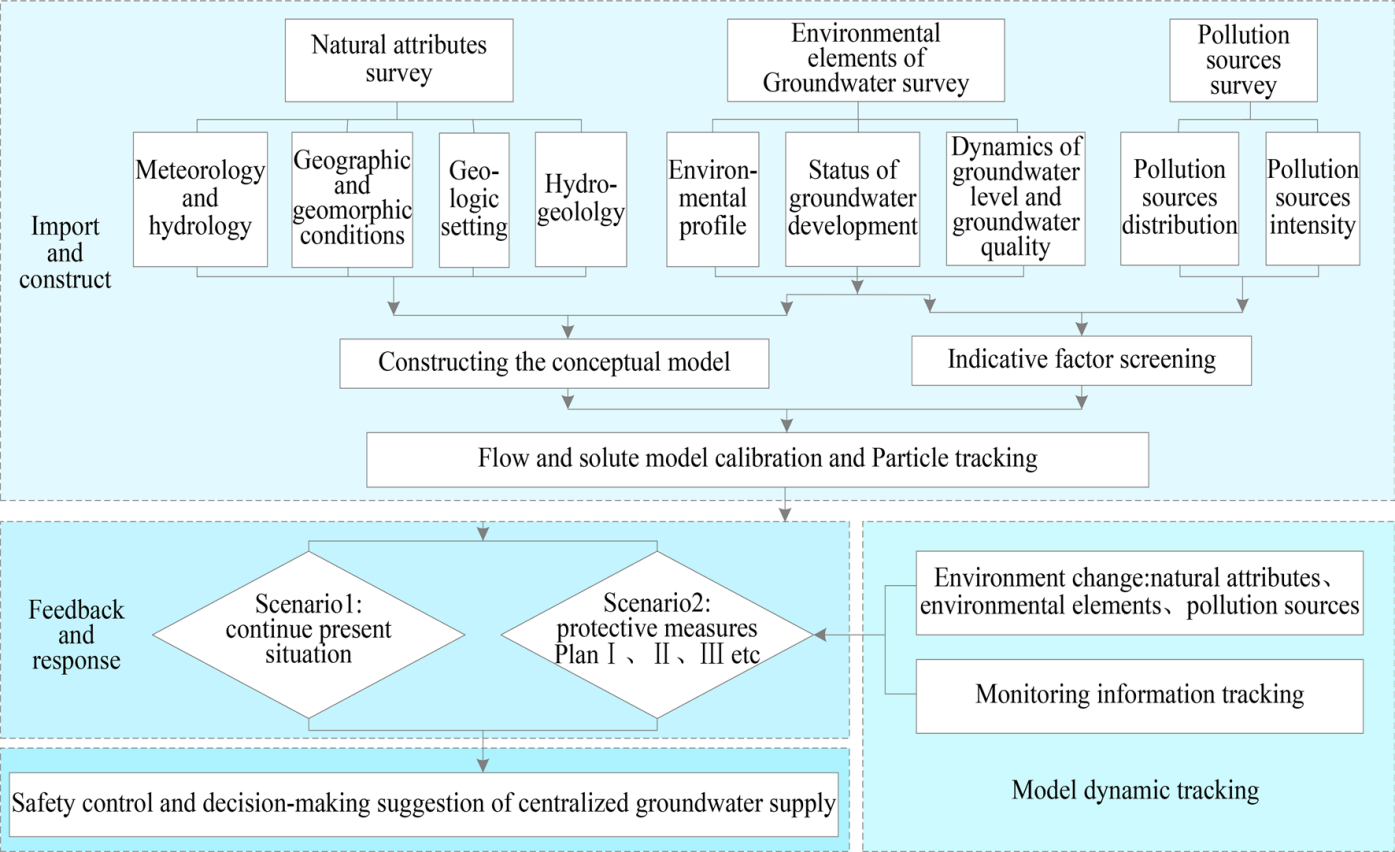
Drinking-groundwater source safety management.

Although there are significant differences in hydrogeological setting of groundwater, most of the unconfined aquifers as centralized drinking-groundwater source are open systems and highly permeable, which results in their high safety risk. Studies had shown that the degree of safety risk can be described by the groundwater vulnerability assessment which describes the probability or tendency of pollutants to reach a specified location after infiltration into a groundwater system and assesses the impact on groundwater function^20,21^. Several studies identifies the spatial distribution of these possible vulnerable zones resulting from the migration of pollutants through a vulnerability prediction model, which are important to protect the groundwater environment and obtain the continuous water supply with high-quality in the region^22,23^. However, such a method is not completely applicable to the safe management of existing groundwater sources in regions where urbanization and industrialization are still immature. The degree of economic development, environmental factors, hydrogeological setting and energy inputs into the environmental protection vary in these regions. Based on these facts, a fresh drinking-groundwater source safety-management system based on a groundwater numerical model with an input–feedback–decision section was developed. The feedback principle in management theory holds that the effectiveness of management depends on a perfect management information system, and accurate, sensitive and powerful information feedback that should also apply to the safe management of groundwater-drinking sources. A perfect information system is constructed by collecting a large amount of basic data that is quantified as a “groundwater model”. The “sensitivity” and “accuracy” of the groundwater model are verified through a comparative analysis of model-calculated and observed values. Figure 4 illustrates that the system construction is divided into four parts: ① The input and construction is the basis of the system, in which the natural properties, the groundwater environment status and pollution source are quantified comprehensively to construct the groundwater conceptual model. ② As a core of the system, a series of remediation measures was proposed and quantified in the model to predict the feedback and response. ③ The predictive model provides a scientific basis to support management decisions. ④ The section of model dynamic tracking is set to achieve a continuous-management system function. When subsequent environmental factors change, the information of drinking-water sources in different periods can be expressed by adjusting the models’ numerical information.

### Sampling and measurement techniques

Groundwater samples were collected after a current-status of groundwater-use survey from 11 representative wells, which included exploitation wells (SW), abandoned tube wells (TW) and bore wells (BW) during July 2015 (Table 1 and Fig. 1). According to the American Public Health Association guidelines, the samples were collected after 10 min of pumping, the volume of pumped water is not less than the volume of sampling well about 0.14m^3^. The samples were stored in polyethylene bottles which were rinsed thoroughly with sampled groundwater at 4 °C. For the accuracy of the test, the pH and electrical conductivity (EC) were measured in the field-use electrode method immediately after sampling. Samples were analyzed in the laboratory to determine their various factors concentration using standard methods as suggested by the American Public Health Association^24^. Table 2 shows the detection method and minimum detectable value of each factor. The total hardness (TH) was determined by the ethylene diamine tetracetic acid (EDTA) titrimetric method. The total dissolved solids (TDS) were measured by weighing. Sulfate, chloride and fluoride were analyzed by ion chromatography. Na, K, Ca, Mg, Fe, Mn, Cu, Zn and Pb were determined by absorption spectrophotometry. Cr^6+^ was determined by diphenylcarbonylhydrazine spectrophotometry. Hg and As were analyzed by atomic fluorescence. COD_Mn_ was determined by spectrophotometry. NH_3_–N was analyzed by the nashi reagent spectrophotometry method. All water-quality parameters have been expressed in milligrams per liter (mg/L), and pH is dimensionless.

**Table 1:** Details of groundwater sampling.

ID	Location	Type of well	Depth	ID	Location	Type of well	Depth (m)
**Groundwater quality monitoring points**							
1#	/	SW	45 m	2#	Distance 1# southeast side 285 m	SW	45
**Groundwater quality and level monitoring points**							
OC3/HW1	Distance 1# north side 1582 m	TW	22 m	OC8/ZK1	Distance 1# northeast side 1190 m	BW	30
OC4/HW6	Distance 1# east side 2926 m	TW	34 m	OC9/ZK2	Distance 1# east side 712 m	BW	25
OC5/HW13	Distance 1# southeast side 3745 m	TW	33 m	OC10/ZK3	Distance 1# east side 213 m	BW	25
OC6/HW15	Distance 1# southwest side 953 m	TW	24 m	OC11/ZK5	Distance 1# northeast side 567 m	BW	25
OC7/HW17	Distance 1# southeast side 2060 m	TW	24 m				
**Groundwater level monitoring points**							
ZK04	Distance 1# southwest side 542 m	TW	18 m	HW9	Distance 1# east side 3352 m	TW	32
HW2	Distance 1# north side 1100 m	TW	25 m	HW10	Distance 1# east side 3826 m	TW	27
HW3	Distance 1# northeast side 2240 m	TW	33 m	HW11	Distance 1# southeast side 4198 m	TW	29
HW4	Distance 1# northeast side 2595 m	TW	29 m	HW12	Distance 1# southeast side 4530 m	TW	22
HW5	Distance 1# northeast side 2163 m	TW	35 m	HW14	Distance 1# southeast side 3623 m	TW	23
HW7	Distance 1# northeast side 3870 m	TW	27 m	HW16	Distance 1# south side 1446 m	TW	19
HW8	Distance 1# east side 4371 m	TW	34 m

**Table 2:** Details of monitoring method and detected minimum.

Number	Monitoring factors	Detection method	Instrument	Minimum detectable value	Instrument sensitivity
1	pH	Electrode method	Portable multi-parameter water quality rapid meter	0~14 (field)	–
2	EC			1μS/cm (field)	–
3	TH	EDTA-2Na titration	–	1.0 mg/L (lab)	–
4	TDS	Weighing method	Sensitive Balance	–	–
5	HCO_3/ Bicarbonate_	titration	–	5 mg/L (lab)	–
6	Sulfate	Ion chromatography	Ion chromatography: ICS-600	0.018 mg/L (lab)	–
7	chloride			0.007 mg/L (lab)	–
8	fluoride			0.006 mg/L (lab)	–
9	Na	Absorption spectrophotometry	Flame/graphite furnace atomic absorption spectrometer: ICE-3500	0.01 mg/L (lab)	Sample volume: 20µL; Cu solution concentration: 20 ng/ml; Absorbance ≥ 0.08Abs
10	K			0.05 mg/L (lab)	
11	Ca			0.5 mg/L (lab)	
12	Mg			0.5 mg/L (lab)	
13	Fe			0.03 mg/L (lab)	
14	Mn			0.01 mg/L (lab)	
15	Cu			0.05 mg/L (lab)	
16	Zn			0.05 mg/L (lab)	
17	Pb			0.001 mg/L (lab)	
18	Cr^6+^	Diphenylcarbonylhydrazine spectrophotometry		0.001 mg/L (lab)	
19	Hg	Atomic Fluorescence	Double beam UV–visible light spectrophotometer: SP-1920	0.00004 mg/L (lab)	Sample: 0.001% potassium dichromate solution; Working conditions:440 nm; Absorbance ≥ 0.01Abs
20	As			0.0003 mg/L (lab)	
21	NH_3_–N	Nashi reagent spectrophotometry	Visible light spectrophotometer: DR-3900	0.025 mg/L (lab)	
22	COD_Mn_	Acid potassium permanganate titration	–	0.05 mg/L (lab)	\

### Groundwater-quality-indicator identification

The standard-index method is a relatively reliable, intuitive and effective method to evaluate the groundwater quality. The calculations are based on standards suggested by the Standards for Groundwater Quality of China^25^. Category 5 grades are classified according to the main functions of groundwater in China. Among them, the Class-III standard expresses the medium content of chemical components in this class of groundwater, which is suitable mainly for drinking, industrial production and agricultural irrigation. According to the current situation of groundwater utilization, the groundwater quality is evaluated based on Class-III standards. The standard-index calculation formula is:

1 $$ P_{i} = \frac{{C_{i} }}{{C_{si} }} $$

where ${P}_{i}$ is the ratio to the standard index of the i-th water-quality factor (dimensionless), ${C}_{i}$ is the monitoring value of the i-th water-quality factor (mg/L) and ${C}_{Si}$ is the Class-III standard value of the i-th water quality factor (mg/L).

When *P*_*i*_ exceeds 1, the groundwater is no longer suitable for drinking, which is attributed to the factor having exceeded the Class-III standard. When Pi value is between 0 and 1, the factor with a *Pi* value much less than 1 (close to 0) mean that the lower risk of contamination without affecting drinking function. The factor with a *Pi* value much higer than 0 (close to 1) mean that the higher risk of contamination, which needs to be monitored and controlled. Such factors can be used as indicators to study the changing trends and management effectiveness of the groundwater environment.

### Equations governing flow and transport processes

Visual Modflow is used widely in various groundwater-contamination transport simulations and to predict the impact of different management plans on pollutant transport in variably saturated heterogeneous regions subject to a variety of boundary conditions^26^. In Visual Modflow, the flow model is modeled by using the modflow module for the three-dimensional (3D) finite-difference numerical simulation of groundwater flow in porous media. This module involves the following partial differential Equation^27^:

2 $$ \frac{\partial }{{\partial x_{i} }}\left( {K_{xx} \frac{\partial h}{{\partial x}}} \right) + \frac{\partial }{\partial y}\left( {K_{yy} \frac{\partial h}{{\partial y}}} \right) + \left( {K_{zz} \frac{\partial h}{{\partial z}}} \right) - W = S_{s} \frac{\partial h}{{\partial t}} $$

where *K*_*xx*_*, K*_*yy*_ and *K*_*zz*_ represent the values of hydraulic conductivity along the *x, y* and *z* coordinate axes, respectively (L T^−1^); *h* is the piezometric head (L); *W* is the volumetric flux per unit volume that is represented for pumping, recharge or other sources, such as sinks (T^−1^); *S*_*s*_ is the specific storage coefficient of the porous material (L^−1^); *t* is the time (T); and *x, y* and *z* are the coordinate directions (L).

The MT3DMS (three-dimensional modular pollutant transport model) module was applied to simulate solute transport in the contaminated aquifer system. The transport model (MT3DMS) uses the flow field generated by the flow model (MODFLOW) to calculate the pollutant plume. In general, solute transport can be described by the following mathematical models^28,29^:

3 $$ \frac{{\partial \left( {\omega C^{k} } \right)}}{\partial t} = \frac{\partial }{{\partial x_{i} }}\left( {\omega D_{ij} \frac{{\partial C^{k} }}{{\partial x_{j} }}} \right) - \frac{\partial }{{\partial x_{i} }}\left( {\omega v_{i} C^{k} } \right) + q_{s} C_{s}^{k} + \sum {R_{n} } $$

where *C*^*k*^ denotes* k* concentrations in water (M L^−3^); ω is the porosity of the porous medium (dimensionless); *t* is time (T); *x*_*i*_ is the distance along the respective cartesian coordinate axis (L); *D*_*ij*_ is the hydrodynamic dispersion cofficient (L^2^ T^−1^); *v*_*i*_ is the seepage or linear porewater velocity (LT^−1^); *q*_*s*_ is the volumetric flux of water per unit volume of aquifer representing sources (positive) and sinks (negative) (T^−1^); *C*_*s*_ is the concentration of sources or sinks (M L^−3^) and ∑ *R*_*n*_ is a chemical reaction term (M L^−3^ T^−1^).

## Physico–chemical characteristics of groundwater and pollution indicator selection

**Figure 5:**
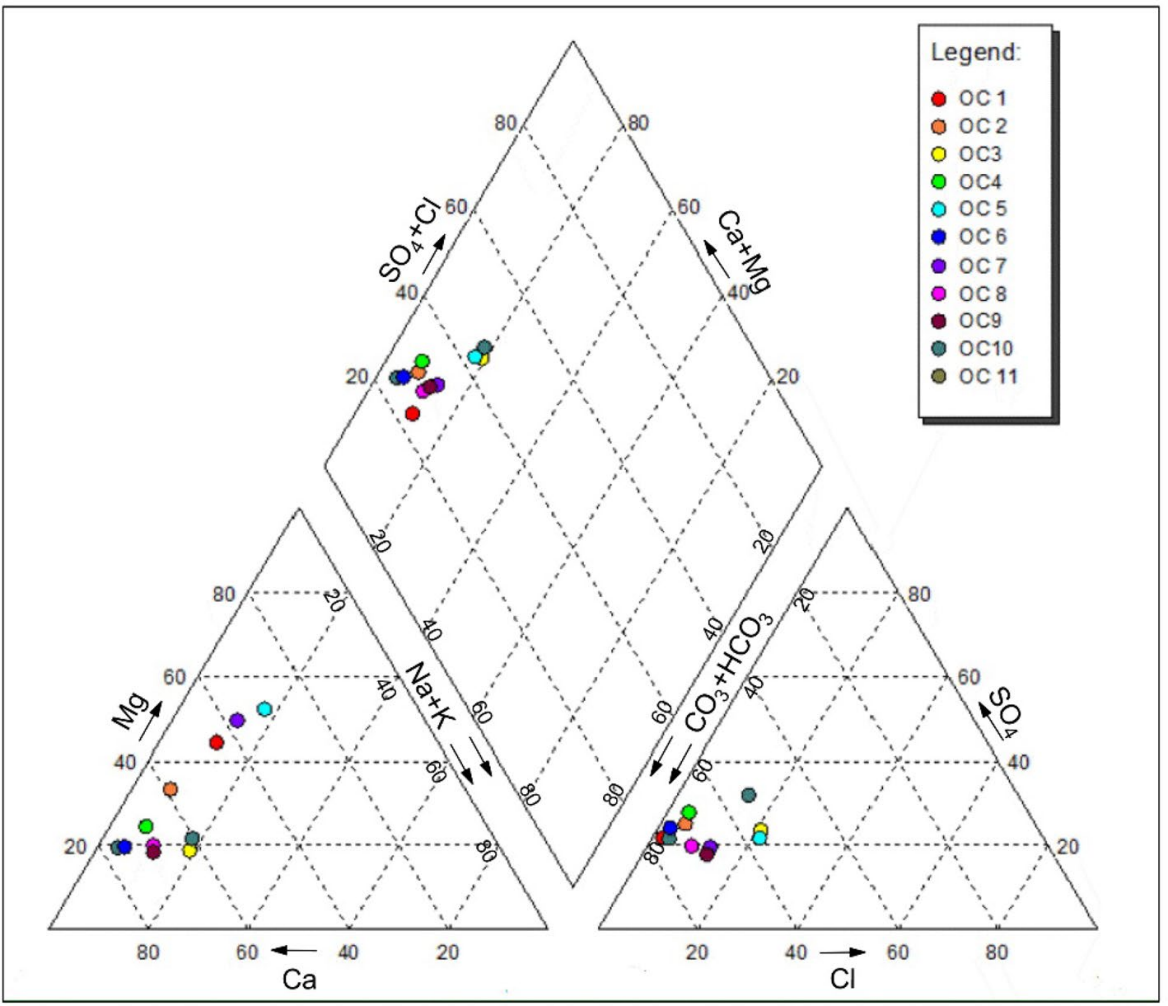
Piper three-line diagram for groundwater-chemical type.

Results from 2015 pre-monsoon groundwater samples are presented in Table 3. The heavy-metal factors such as Fe, Mn, Cu, Zn, Pb and Cr^6+^ did not reach the minimum level of detection without being focused, which is consistent with the characteristics of this type of pollution source in the study area. Na, K, Ca, Mg and bicarbonate are not set to standard values in GB/T14848-2017, which are used to analyze groundwater-chemical types by drawing a Piper three-line diagram. Figure 5 indicates that the main groundwater-chemical type is Ca–Mg–HCO_3_ and is combined with a lower-level TDS concentration (228.0–736.8 mg/L), which indicates that the hydraulic alternating condition is excellent in an alluvial aquifer. The pebble-based structure of the aquifer and the hydraulic conductivity of 10–40 m/day provided by hydrogeological tests also support this judgment.

**Table 3:** Hydrochemical and quality characteristics of water.

Parameters	Groundwater				Surface water (Fu jiang River)				II Permissible
	Min	Max	Average	P_i_	Min	Max	Average	P_i_	
TDS	222.80	736.80	452.55	0.22~0.74	171.39	185.35	177.76	0.17~0.18	1,000
EC	318.29	1,270.34	665.51	–	239.946	324.3625	279.0832	–	–
COD_Mn_	0.74	2.78	1.19	0.25~0.93	0.88	0.99	0.92	0.29~0.33	3.0
NH_3_–N	0.02	0.95	0.19	0.04~1.90	0.03	0.08	0.05	0.06~0.16	0.5
TH	121.50	370.90	301.04	0.27~0.82	135.42	164.87	155.23	0.30~0.36	450
Fluoride	0.10	0.30	0.18	0.10~0.30	0.11	0.12	0.12	0.11~0.12	1
Na	7.00	50.00	22.48	0.04~0.25	19.23	24.35	22.56	0.10~0.12	200
K	0.50	2.90	1.52	–	0.70	2.40	1.37	–	–
Ca	40.08	146.30	93.37	–	104.56	118.02	110.64	–	–
Mg	13.38	62.02	27.36	–	16.24	18.85	17.75	–	–
Bicarbonate	170.90	384.40	312.31	–	317.53	332.94	326.78	–	–
Chloride	3.55	76.23	31.65	0.01~0.30	23.05	38.29	29.88	0.09~0.15	250
Sulfide	48.48	180.00	88.52	0.19~0.72	68.73	72.48	72.32	0.28~0.29	250
Fe	ND	ND	ND	–	ND	ND	ND	–	0.3
Mn	ND	ND	ND	–	ND	ND	ND	–	0.1
Cu	ND	ND	ND	–	ND	ND	ND	–	1
Zn	ND	ND	ND	–	ND	ND	ND	–	1
Hg	ND	ND	ND	–	ND	ND	ND	–	0.001
As	ND	ND	ND	–	ND	ND	ND	–	0.01
Cd	ND	ND	ND	–	ND	ND	ND	–	0.005
Cr^6+^	ND	ND	ND	–	ND	ND	ND	–	0.05
Pb	ND	ND	ND	–	ND	ND	ND	–	0.01

TDS is related directly to the concentration of ionized substances in groundwater and may also be related to problems of excessive other-mineral contamination, such as EC and TH. Among the detected factors, the concentration of NH_3_–N in OC11 reached 0.95 mg/L, which means that it exceeded the Class-III standard. OC11 is located 567 m upstream of the northeast side of the exploitation wells and 980 m downstream of the southwest side of the point pollution P3-1, which is the closest monitoring point downstream of industrial pollution sources The *Pi* of a surplus-detected factor for all samples was less than 1. In the study area, the groundwater pH ranged from 6.8 to 7.5. The COD_Mn_ ranged from 0.74 to 2.78 mg/l. The NH_3_–N varied from 0.02 to 0.95 mg/l. The concentration of fluoride varied from 0.1 to 0.3 mg/l. The concentration of chloride varies from 3.55 to 76.23 mg/l. The concentration of sulfide in groundwater varied from 48.48 to 180.0 mg/l. Based on this monitoring data, the detected factor values of the 1# and 2# samples were within the scope of the Class-III standard and could be used for various purposes, including drinking. In addition, Fujiang River is an important source of supply for wells 1 # and 2 #. In this study, three sections of Fujiang River was also monitored, the sampling sections are shown in Fig. 1. The monitoring results show that the factors in the surface water samples are far below the Class-III standard, which means that recharge of Fujiang River will not influence the groundwater drinking function (Table 3).

**Figure 6:**
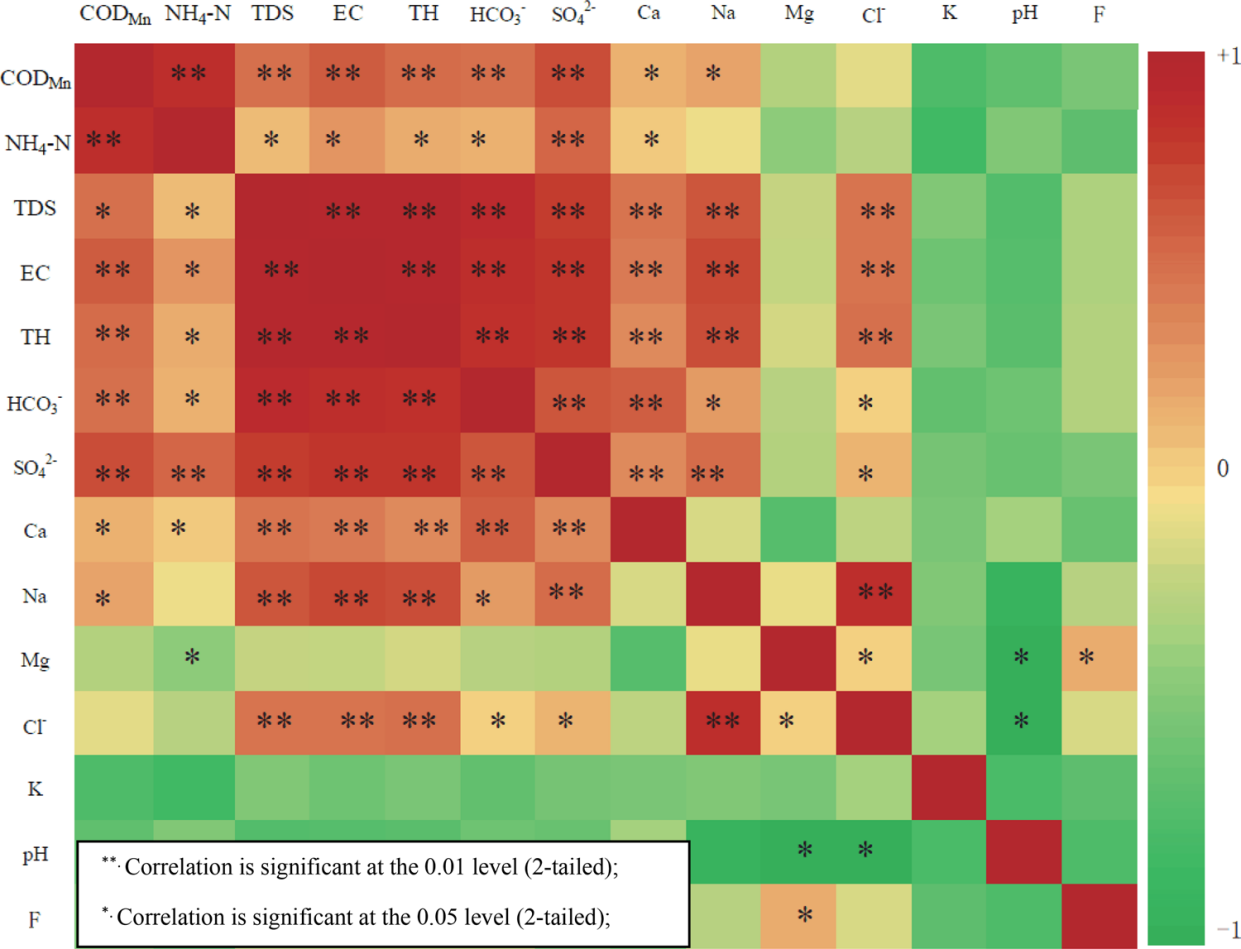
Correlation heat map between factors.

The significant concentration fluctuation in the same alluvial aquifer with excellent hydraulic alternating conditions indicates the impact of exogenous substance input on subsurface environment. COD_Mn_ and NH_3_–N as typical factors are weakly correlated with the interaction intensity of water and rock, and their concentration fluctuation results mainly from the intervention of anthropogenic activities. COD_Mn_ and NH_3_–N are the typical pollutants in agricultural non-point source pollution, domestic sewage and most industrial wastewater. Both factors with a high *Pi* means that its concentration is close to the standard, which reflects more directly the risk of groundwater contamination. If their representative is verified, this study attempts to use COD_Mn_ and NH_3_–N as indicators for groundwater-drinking safety management. The representativeness of COD_Mn_ and NH_3_–N that reflect the groundwater-quality trend can be analyzed by the person correlation-analysis method through SPSS software. The original 8.0 was used for mapping (Fig. 6).The correlation between COD_Mn_, NH_3_–N, TDS, EC, TH, F, Na, K, Ca, Mg, bicarbonate, chloride and sulfide in 11 samples was analyzed. Significant acid–base and fluoride-pollution sources were not found, so the fluctuations of pH and F were inconspicuous and there was no significant correlation between COD_Mn_ and NH_3_–N. Figure 6 shows that the COD_Mn_ and NH_3_–N have a medium–high correlation with TDS, TH, EC and other factors with a high concentration in groundwater, such as Na, Ca, bicarbonate and sulfide etc., and the correlation coefficients are 0.774–0.842, 0.593–0.712, 0.584–0.789, 0.551–0.872, respectively. According to the above analysis, COD_Mn_ and NH_3_–N are suitable as indicators, and their fluctuation concentrations can reflect the variation characteristics of groundwater quality in the study area.

## Flow and contaminant-transport modelling

### Model construction

#### Model grid and parameterization

The simulated model domain of the groundwater flow model of Fujiang River watersheds that cover approximately 25 km^2^ has been constructed using the above hydrogeological database. Quaternary alluvial and bedrock are divided naturally into aquifers and aquitard because of the significant difference in hydraulic conductivity. Field geological observations showed that the thickness of the quaternary alluvial aquifer was between 35 and 40 m. A uniform grid spacing of 50 m × 50 m was used in the model. The model uses the MODFLOW-2000 module to simulate the groundwater flow^27^.

**Figure 7:**
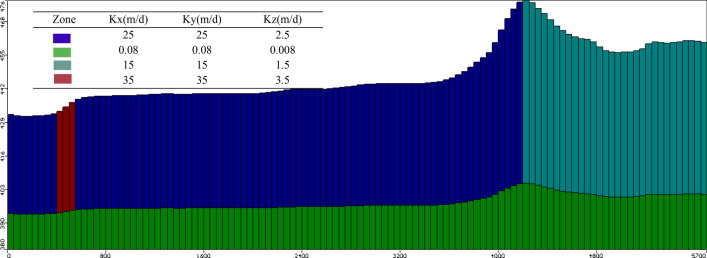
Spatial-distribution hydraulic conductivity.

Certain hydrogeological parameters are indispensable for the establishment of hydrogeological numerical models of the studied site. Detailed information collection, geological investigation and in-situ hydrogeological tests provide the necessary inputs for modeling, such as hydraulic conductivity, specific storage, dispersion coefficient, effective porosity and total porosity. The values of various parameters were inputted to a fitted reasonable model that are shown in Table 4 and the values of hydraulic conductivities (K) of each layer are shown in Fig. 7.

**Table 4:** Parameter values used in numerical model.

Model parameter	Value
Length of model domain in *x*-direction/m	5700
Length of model domain in *y*-direction/m	5400
Active simulation area/km^2^	25.08
Dimension of one grid cell/m	50 * 50
Rainfall infiltration coefficient	0.10~0.18
Specific yield (Sy)	0.2
Effective porosity	0.2
Total porosity	0.3
Specific storage, (Ss/m)	1.0 * 10^–7^
*D*_L_ longitudinal dispersion	0.467
*D*_T_ Ratio of longitudinal dispersion to transverse dispersion	1/10D_L_

#### Boundary conditions

**Figure 8:**
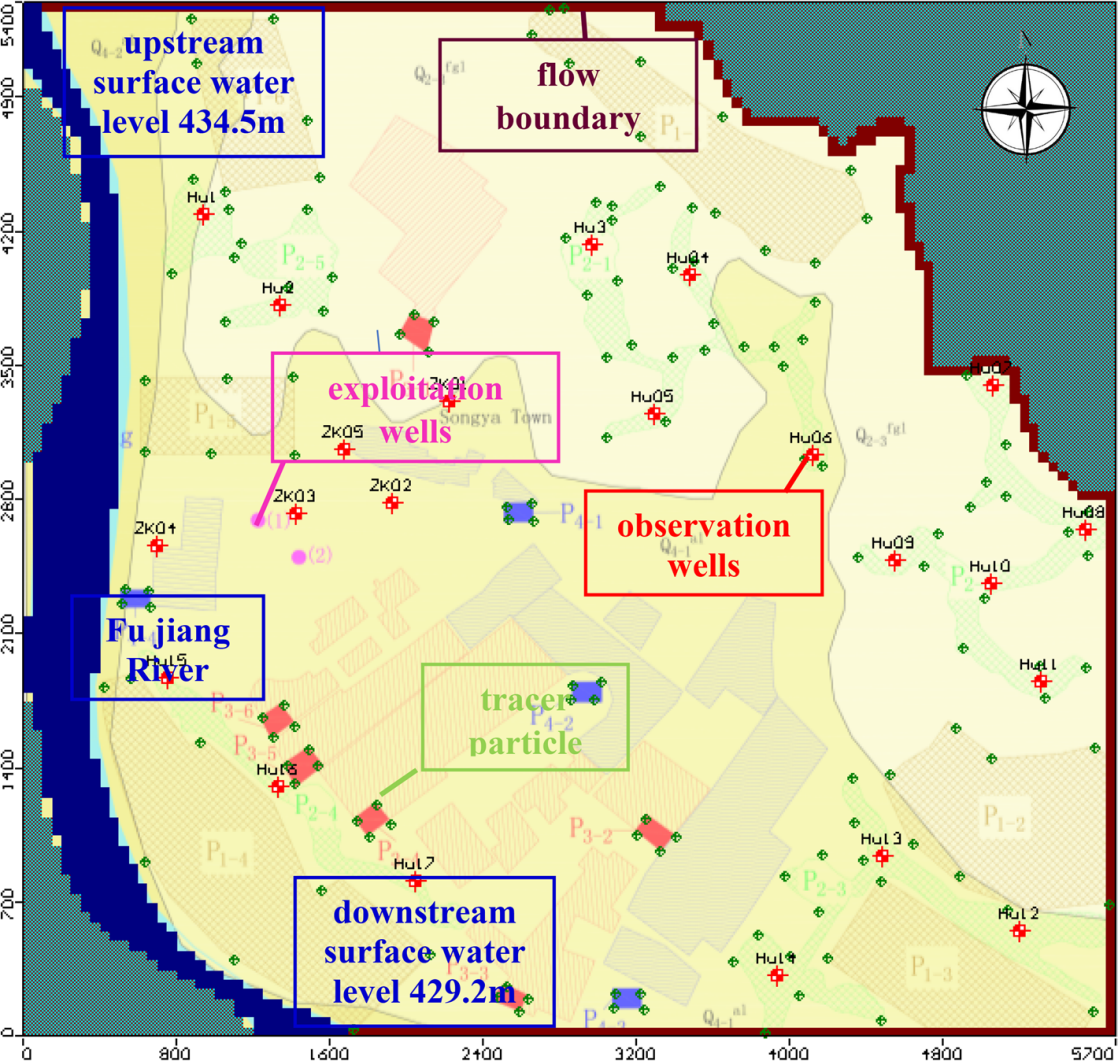
Positions of boundary, tracer particle and observation wells of the study area. The map was generated by Visual Modflow version 4.0. The URL of the source map is https://geocloud.cgs.gov.cn/#/portal/geologicalDatabase/ArealGeology?type=dzsjk.

For the groundwater flow model, the boundary conditions describe the flow exchange between the model and the external system^30^. The objectivity and accuracy of the boundary condition setting determine the possibility of the simulated solution and the rationality of the model. Based on the knowledge of the conceptual model of the simulation zone, the boundary settings are as follows. The western boundary Fujiang River was considered a constant head for this study area. The northeast side of the bedrock mountain area does not contribute significantly to the aquifer recharge, so sets the zero flow boundary. The northern and eastern sides are upstream of the groundwater runoff direction of the study area, so they were considered as the hydrological flow boundary (Fig. 8). The south side is downstream of the simulation area and is set to the outflow boundary. The recharge of the study area is mainly atmospheric precipitation. The annual average rainfall is 919 mm and the rainfall infiltration coefficient ranges from 0.15 to 0.18. A manual exploitation of groundwater is an important discharge mode. Therefore, two groundwater pumping wells were set in the model, with capacities of 10,000 m^3^/day (1#) and 5000 m^3^/day (2#). Moreover, four types of 21 pollution sources were distributed around the exploitation wells, 70 tracer particle points were set to identify the pollution source that may affect the quality of the groundwater supply in the model. The model domain with the positions of the observation wells, tracer particle points and boundary conditions is shown in Fig. 8.

#### Pollution source setting of transport model

MT3DMS is a computer model that is used to simulate advection, dispersion and chemical reactions of contaminants in three-dimensional groundwater flow systems^28,31^. In the previous analysis, COD_Mn_ and NH_3_–N were selected as indicator factors for groundwater solute-transport simulation. The initial values of COD_Mn_ and NH_3_–N were set to the lowest detected values of 0.74 mg/L and 0.02 mg/L, respectively. In the study area, the highest concern regarding groundwater pollution was focused on the contamination associated with anthropogenic activities. Different pollution fluxes were assigned to the model according to the pollution**-**source investigation (Tables 5 and 6).

**Table 5:** Non-point-source pollution statistics.

Number	Type	Area (km^2^)	Pollution intensity (kg/day ·km^2^)	
			NH_3_-N	COD_Mn_
P_1-1_	Agricultural non-point source (Greenhouse vegetable cultivation)	0.654	1.03~1.64	2.26~3.61
P_1-2_		0.851		
P_1-3_		0.571		
P_1-4_		1.217		
P_1-5_		0.217		
P_1-6_		0.420		
P_2-1_	Unorganized emission	0.435	0.82~1.31	2.06~3.30
P_2-2_		0.432		
P_2-3_		0.530		
P_2-4_		0.229		
P_2-5_		0.311

**Table 6:** Point-source pollution statistics.

Number	Type	Main body structure size (L × B × H)	Pollution flux (kg/day)	
			NH_3_-N	COD_Mn_
**Industrial point-pollution**				
P_3-1_	Liquor brewing industry	40 × 30 × 2.5	33.75~101.25	112.5~337.50
P_3-2_		38 × 25 × 3.0		
P_3-3_	Chinese medicine preparation industry	40 × 22 × 2.5		
P_3-4_		36 × 20 × 2.0		
P_3-5_	Mechanical processing industry	55 × 30 × 2.3		
P_3-6_		45 × 32 × 2.5		
P_4-1_	Point-pollution source of central residential	15 × 10 × 3	4.87~14.63	14.62~43.88
P_4-2_		12 × 8 × 3		
P_4-3_		12 × 8 × 3		
P_4-4_		15 × 10 × 3		

Non-point-source pollution flux is defined as the product of pollution intensity and the area of the source. In the agricultural non-point-source areas, the pollution-emission intensity of COD_Mn_ was between 2.26 and 3.61 kg/day ·km^2^, and the NH_3_–N was between 1.03 and 1.64 kg/day ·km^2^. In the unorganized emission areas, the pollution-emission intensity of COD_Mn_ was between 2.06–3.30 kg/day ·km^2^, and the NH_3_–N was between 0.82–1.31 kg/day ·km^2^^8,32^. Six industrial point-pollution source (P3-1–P3-6) and four central residential point-pollution sources (P4-1–P4-4) exist in the study area. Point source pollution is mainly pool structures, and the volume of sewage infiltration is calculated based on the Darcy seepage mechanism. Point-source pollution flux is defined as the product of infiltration volume and pollution concentration. The NH_3_–N emissions flux from the industrial sewage treatment plants ranged from 33.75 to 101.25 kg/day, and the COD_Mn_ emissions flux ranged from 112.5 to 337.50 kg/day. The NH_3_–N contaminant flux from the domestic sewage pretreatment systems ranged from 4.87 to 14.63 kg/day, and the COD_Mn_ emissions ranged from 14.62 to 43.88 kg/day. The flux of each pollution source was adjusted to calibrate the solute-transport model by trial and error.

### Flow-model calibration

**Figure 9:**
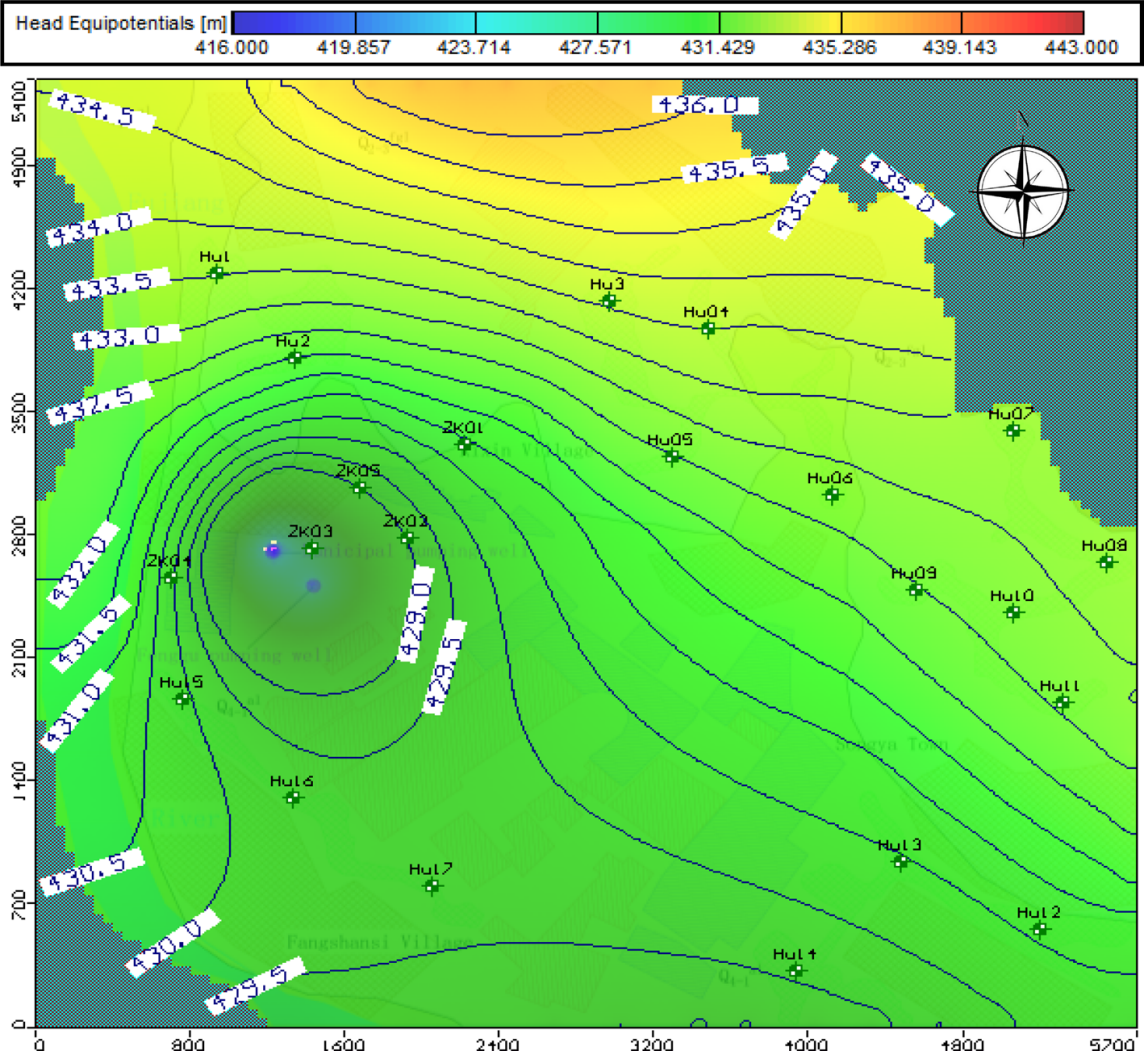
Equipotential lines and vectors that indicate the flow direction and magnitude of underground flow. The map was generated by Visual Modflow version 4.0. The URL of the source map is https://geocloud.cgs.gov.cn/#/portal/geologicalDatabase/ArealGeology?type=dzsjk.

In this study, the groundwater model assumed that the basic structure, such as aquifer thickness, model mesh size and boundary conditions are known and invariant. Based on this assumption, uncertain parameters such as hydraulic conductivity, recharge, and specific yield are adjusted to output a series of simulation models that are compared with actual monitored values to complete model calibration by trial and error. The rationality and accuracy of the model was judged by comparing the calculated mean absolute (MA) and root-mean-squared (RMS) error^33^. As shown in Fig. 9 that 22 actual observed wells which ordered distribution and covering the study area were used for the flow model verification. The data statistics in Table 7 shown that the RMS error and MA is small, just 0.43 m and 0.33 m, respectively, and the calculation of calibrated model versus observed hydraulic heads matched closely. The hydraulic conductivity of the partition setting is shown in Fig. 6 and the specific yield and recharge were set to 0.15 and 77 mm/a in the calibrated model, respectively. Figure 9 data indicate that the maximum flow velocity was 5.5 m/day and a drop funnel with a maximum depth of 12 m resulted because of groundwater exploitation in the flow field. A solute-transport simulation will be based on a calibrated flow model.

**Table 7:** Computed versus observed head values.

Well/point bame	Obs	Calc	Calc.-Obs	Well/point name	Obs	Calc	Calc.-Obs
HW1/A	433.54	433.52	− 0.02	HW12/A	431.12	430.58	− 0.54
HW2/A	432.15	431.97	− 0.18	HW13/A	431.39	430.47	− 0.92
HW3/A	434.36	434.08	− 0.28	HW14/A	429.35	429.57	0.22
HW4/A	433.87	433.99	0.12	HW15/A	431.17	430.1	− 1.07
HW5/A	432.27	432.48	0.21	HW16/A	429.56	429.72	0.16
HW6/A	432.48	432.73	0.25	HW17/A	429.13	429.63	0.5
HW7/A	433.77	433.34	− 0.43	ZK01/A	431.01	430.89	− 0.12
HW8/A	433.24	433.21	− 0.03	ZK02/A	429.29	429.04	− 0.25
HW9/A	432.36	432.46	0.1	ZK03/A	426.62	425.88	− 0.74
HW10/A	432.37	432.75	0.38	ZK04/A	430.35	430.12	− 0.23
HW11/A	432.11	432.58	0.47	ZK05/A	428.95	428.97	0.02
MA				0.33			
RMS				0.43			

### Particle-tracking model analysis

A particle-tracking method associates the flow field with an imaginary particles in the model, and simulates the migration path of the particles in the groundwater flow model at sequential intervals of flow time. The shape of the flow paths were affected by the hydraulic gradient and flow pattern and the particles setting. The MODPATH module based on this approach is applied to track the migration path of particles (forward approach), identify potential sources of pollution (backward approach) and assess groundwater environmental vulnerability^34–36^. To identify the potential source of pollution that may impact the groundwater supply quality, 70 tracer particles were set up in the model that covers all sources of pollution in a spatial distribution based on pollution-source investigation.

**Figure 10:**
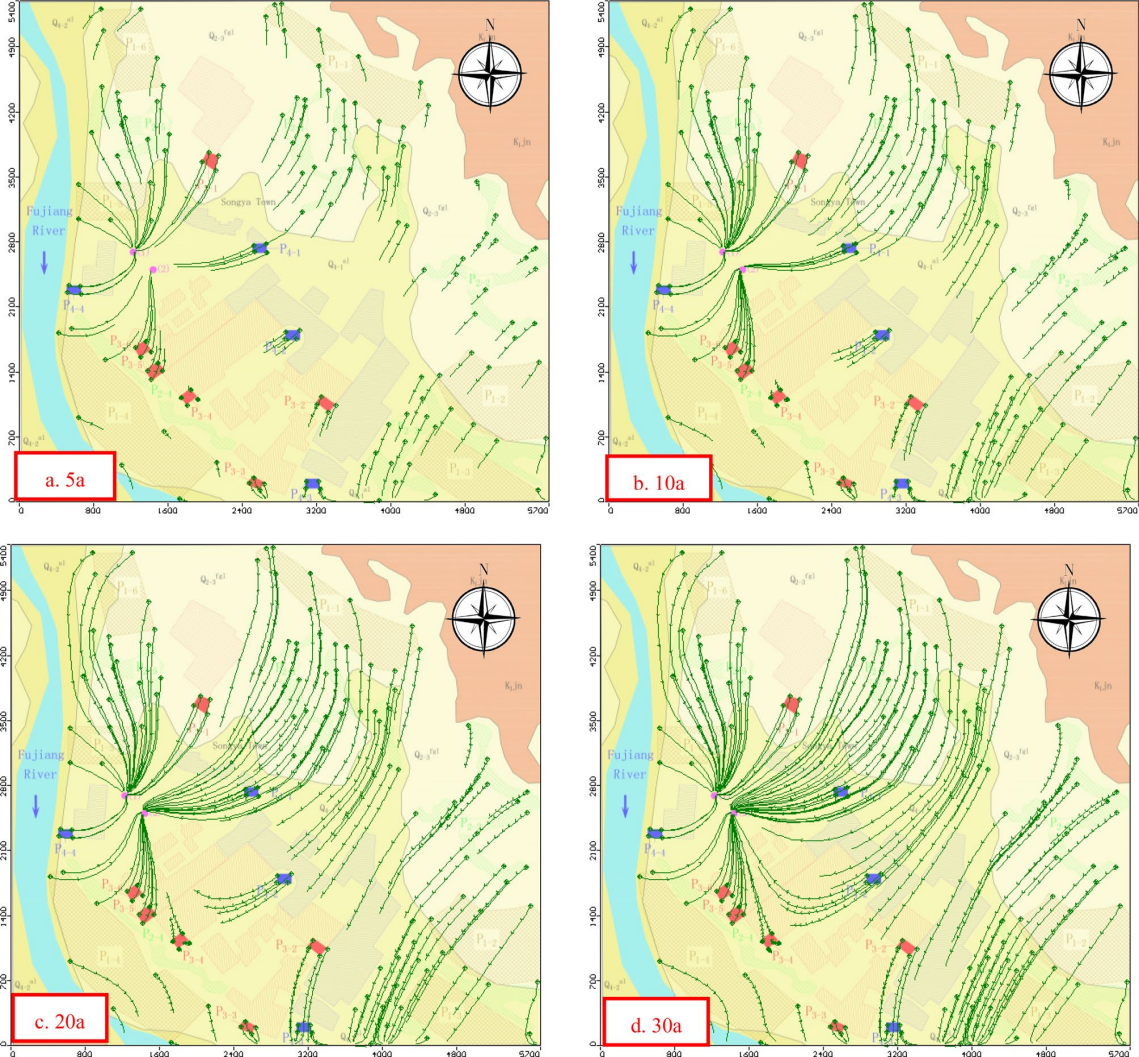
Forward calculation of tracer-particle migration path. The map was generated by Visual Modflow version 4.0. The URL of the source map is https://geocloud.cgs.gov.cn/#/portal/geologicalDatabase/ArealGeology?type=dzsjk.

The capture zones of the tracer particles for 30 years was forward calculated based on a calibrated flow model. Figure 10 shows the relationship of the path of the tracer particles to the exploitation wells versus time. Figure 10c shows that the tracer particles of all the potential pollution sources have been transported to the production well after 20 years of operation. The drop funnel formed by groundwater pumping has rendered the original upstream–downstream relationship no longer significant. The most obvious characteristic of the flow field was that the drainage base level and the aquifer downstream of the south side were transformed to recharge the exploitation wells. The particle-capture zone in the downstream direction of the south side extends to 1,100 m. The variation in direction of the groundwater runoff increases the risk of migration of the pollution sources from different directions to the groundwater supply source. Moreover, the increased hydraulic gradient increases the migration rate and flux of the contaminants, because of the drop funnel. According to the results in Fig. 10, the sources of pollution that may affect the quality of the groundwater supply include: P1-1, P1-3, P1-4, P1-5 and P1-6 in an agricultural non-point source; P2-1, P2-4 and P2-5 in the source of non-organized emission of the dispersed population; P3-1, P3-4, P3-5 and P3-6 in the industrial sewage-treatment plant and finally; and P4-1, P4-2 and P4-4 in the pretreatment system for centralized domestic sewage.

### Transport model calibration

To complete this study, a solute-transport model for judging major sources of pollution was used to formulate and analyze control measures of potential contamination sources in the drinking-groundwater source after the groundwater model and particle tracking had been described. The trial-and-error method is applicable to the calibration of the seepage field model and to the calibration of the transport model^33^. According to the mathematical equation of solute transport, the uncertainty parameters that affect the transport model include the diffusivity and the infiltration flux of the contaminant based on the calibrated flow field. The uncertainty parameter variable is adjusted and the model calculation value is compared with the actual monitoring value of the indicator factor to complete the calibration.

**Figure 11:**
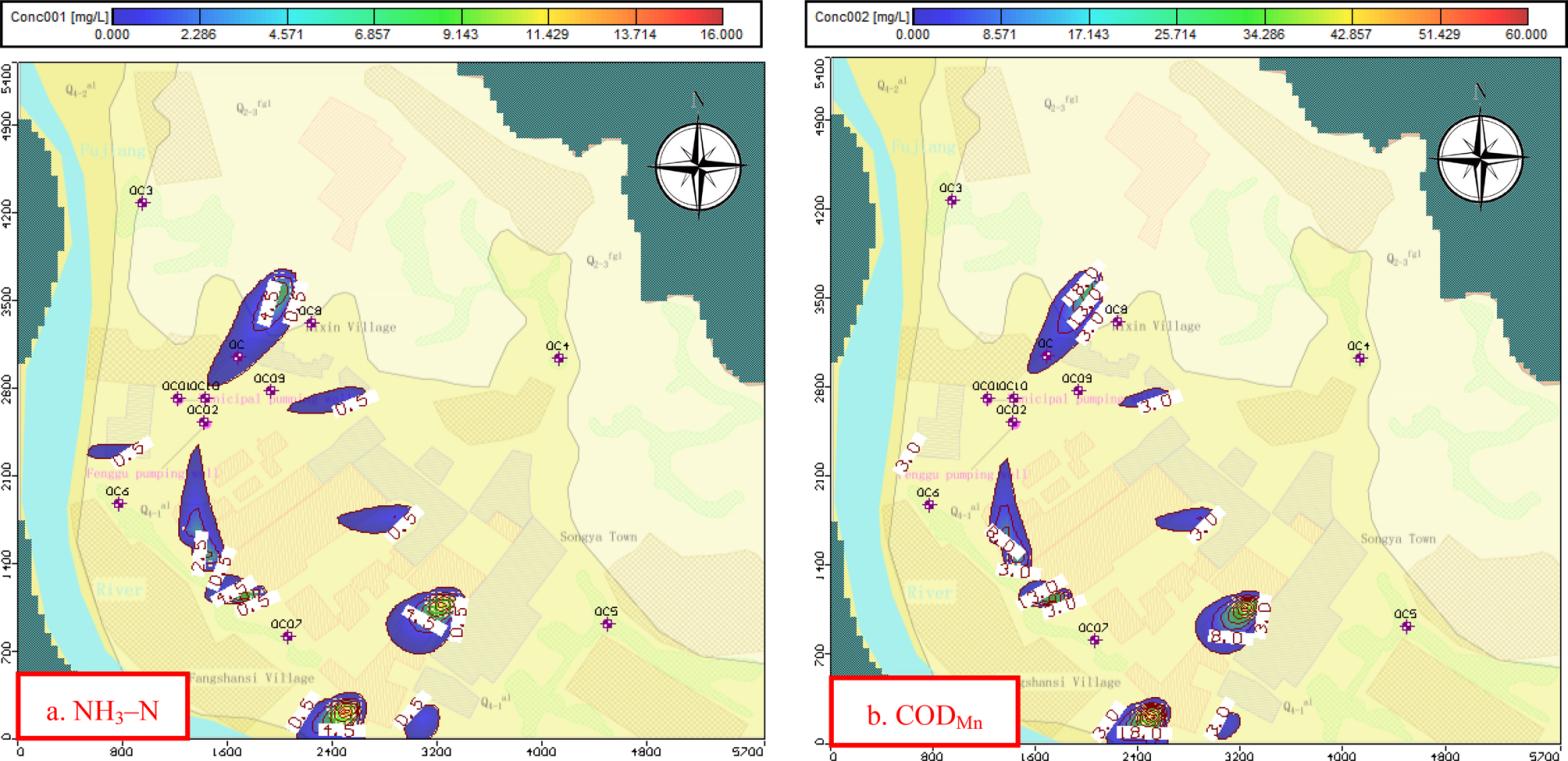
Pollutant concentration distribution after calibration. The map was generated by Visual Modflow version 4.0. The URL of the source map is https://geocloud.cgs.gov.cn/#/portal/geologicalDatabase/ArealGeology?type=dzsjk.

**Figure 12:**
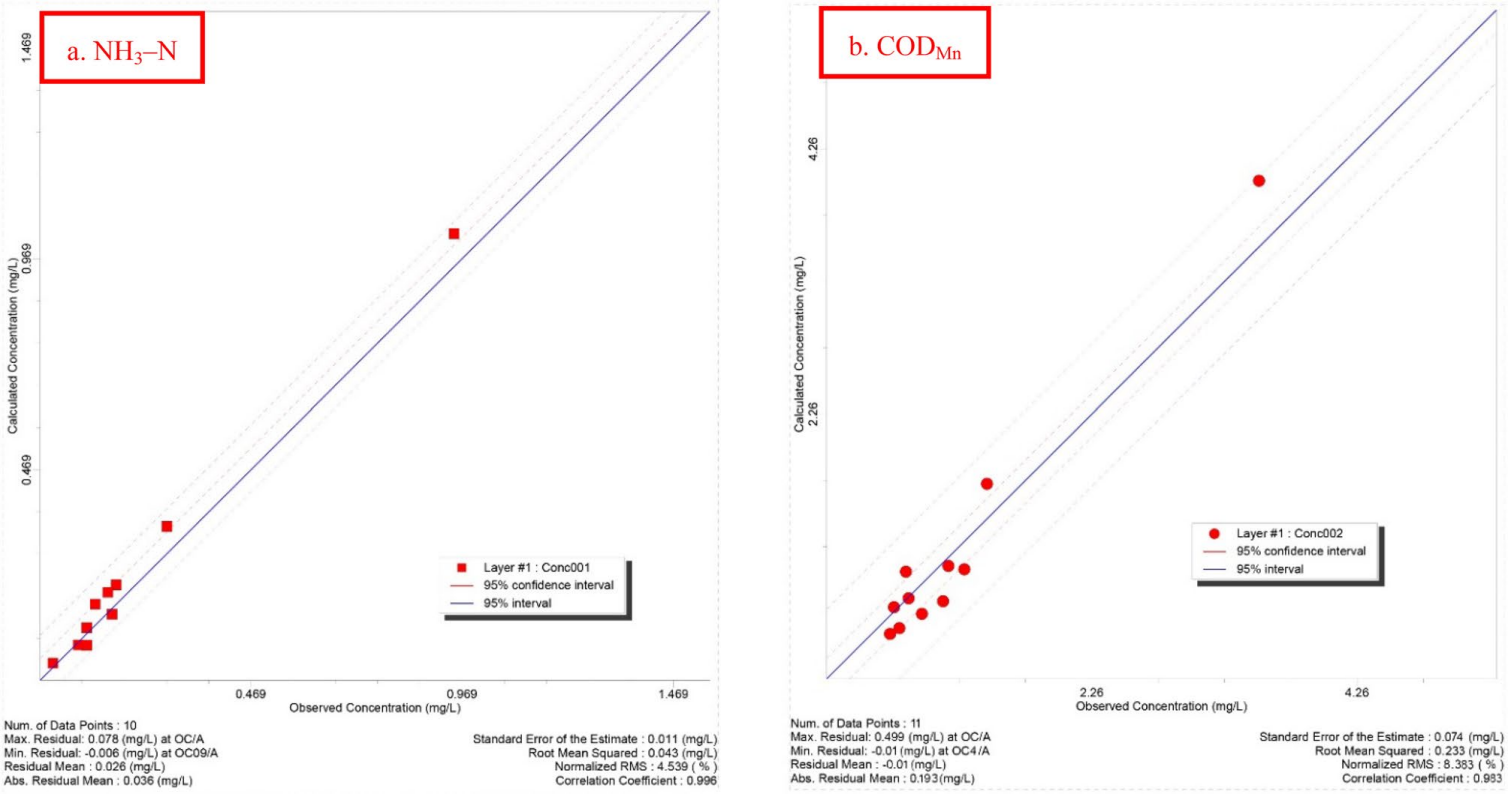
Calculated versus observed concentration.

As shown in Fig. 11 that 11 actual concentration observed wells were used for the transport model verification. After calibrating the solute-transport model, the RMS errors of COD_Mn_ and NH_3_–N were 0.233 and 0.043 mg/L, respectively. The absolute residual mean values were 0.193 mg/L and 0.036 mg/L, respectively (Fig. 12). The area where groundwater quality did not conform to the Class-III standard because of excessive NH_3_–N and COD_Mn_ concentrations closest to the 1# and 2# groundwater exploitation wells was 240 m (Fig. 11).

The above discussion indicates that expanding the scope of recharge because of groundwater exploitation increases the risk of groundwater contamination. According to the results in Fig. 12, the most important impact on the deterioration of groundwater quality is the industrial pollution source, and the emission of concentrated residents is second among the four types of pollution sources. The migration mode of non-point pollution source is infiltration into groundwater through precipitation leaching. When the recharge of the exploitation wells includes intensified recharge from the aquifer and surface water, the non-point pollution source with a relatively small flux has no obvious impact on water quality because of dilution. A focus on the safe management of groundwater-drinking water sources is the remediation of the point pollution sources.

## Prediction results and discussion

The calibrated model was run to predict different scenarios up to a period of 20 years, i.e., from June 2015 to June 2034. To analyze the necessity and effectiveness of remediation measures for the safety of drinking-groundwater sources, two scenarios were considered to predict the concentration of NH_3_–N and COD_Mn_ in the groundwater exploitation wells over 20 years.

**Figure 13:**
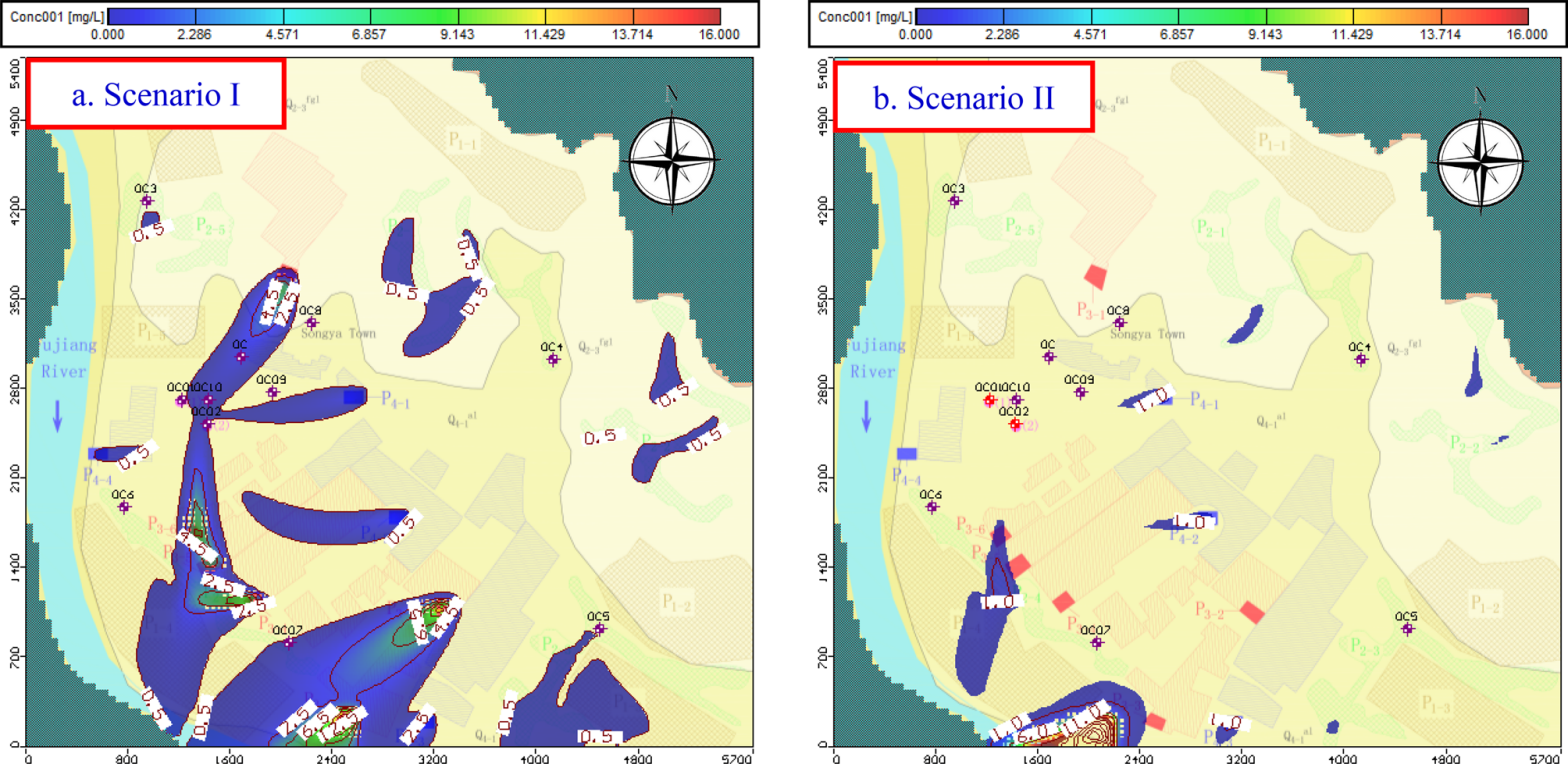
Variation in concentration of NH_3_–N at the end of 2035. The map was generated by Visual Modflow version 4.0. The URL of the source map is https://geocloud.cgs.gov.cn/#/portal/geologicalDatabase/ArealGeology?type=dzsjk.

Scenario I (Fig. 13a): This scenario determined the effect on groundwater quality after 20 years if no remediation measures have been taken. Figure 12a shows that the pollution plume generated by P3 and P4 pollution sources continued to migrate to the exploitation wells, and the concentration of NH_3_–N and COD_Mn_ in the groundwater exploitation wells continued to increase annually. The concentration of COD_Mn_ in 1# exploitation well rose to 1.03 mg/L after 5 years of operation (2020a), and continued to stabilize until the end of the prediction period. The NH_3_–N concentration continued to increase to 0.30 mg/L until the end of the prediction period. The concentration of COD_Mn_ in the 2# exploitation well continued to increase to 1.96 mg/L until the end of the prediction period. The NH_3_–N concentration increased to 0.5 mg/L after 16 years of operation (2031a), which exceeded the Class-III standards in GB/T14848-2017, and then continued to increase to 0.54 mg/L until the end of the forecast period. Based on scenario I in which no precautions and remediation measures were taken, the water quality of 2# will be affected significantly and will no longer be suitable for drinking.

Scenario II assumed that the following remedial measures were taken: (1) At 1,300 m on the south side of the 1# and 2# exploitation wells outside the particle-capture zone, a centralized industrial wastewater treatment plant (P3-7) was built to replace the original treatment station to treat industrial sewage with strict anti-seepage measures. (2) The anti-seepage system of domestic sewage pretreatment facilities were improved such that the infiltration flux was reduced to 50%. According to the prediction, the concentration of indicator factor in the 1# and 2# exploitation wells peaked at 3a after implementation of the measures. The peak concentrations of NH_3_–N were 0.22 and 0.26 mg/L and the COD_Mn_ were 0.92 and 1.33 mg/L, respectively. The concentration of NH_3_–N was stable at 0.13 and 0.19 mg/L, and the concentration of COD_Mn_ was stable at 0.41 and 0.84 mg/L, respectively, by the end of the forecast period (2035a). As exhibited by Fig. 13b, the groundwater quality of the exploitation wells did not exceed the Class-III standard and the drinking function was unaffected.

## Conclusions

This study indicates the increased safety risks of drinking-water sources because of a variation in the surrounding environment that is caused by anthropogenic activities. The change of flow direction which is caused by groundwater exploitation is a non-ignorable control factor during potential pollution source identification. A prevention of this risk should focus primarily on the management and control of industrial pollution sources. The main contribution of a higher concentration of NH_3_–N and COD_Mn_ originates from industrial pollution sources in the study area. The comparative scenario of the model illustrates that the rational industrial layout that considers hydrogeological characteristics and the necessary anti-seepage measures of point pollution can protect the safety of drinking-groundwater sources. The drinking-groundwater source safety-management system was constructed based on a numerical simulation method that can be regarded as the technical support of groundwater-source environmental management. A three-dimensional hydrogeological model has an objective interpretation of contaminant migration and control factors in the aquifer system that can also include quantified prior data, and current and future information in the time dimension. Therefore, the system can solve the current drinking-water-source safety problem, and adjust the quantitative information to provide corresponding decision-making suggestions in the subsequent management process when the environment varies in the future.
